# Prediction of lymph node metastasis in patients with breast invasive micropapillary carcinoma based on machine learning and SHapley Additive exPlanations framework

**DOI:** 10.3389/fonc.2022.981059

**Published:** 2022-09-15

**Authors:** Cong Jiang, Yuting Xiu, Kun Qiao, Xiao Yu, Shiyuan Zhang, Yuanxi Huang

**Affiliations:** Department of Breast Surgery, Harbin Medical University Cancer Hospital, Harbin, China

**Keywords:** machine learning, SHAP, IMPC, nomogram, lymph node metastasis

## Abstract

**Abstract:**

Background and purpose: Machine learning (ML) is applied for outcome prediction and treatment support. This study aims to develop different ML models to predict risk of axillary lymph node metastasis (LNM) in breast invasive micropapillary carcinoma (IMPC) and to explore the risk factors of LNM.

**Methods:**

From the Surveillance, Epidemiology, and End Results (SEER) database and the records of our hospital, a total of 1547 patients diagnosed with breast IMPC were incorporated in this study. The ML model is built and the external validation is carried out. SHapley Additive exPlanations (SHAP) framework was applied to explain the optimal model; multivariable analysis was performed with logistic regression (LR); and nomograms were constructed according to the results of LR analysis.

**Results:**

Age and tumor size were correlated with LNM in both cohorts. The luminal subtype is the most common in patients, with the tumor size <=20mm. Compared to other models, Xgboost was the best ML model with the biggest AUC of 0.813 (95% CI: 0.7994 - 0.8262) and the smallest Brier score of 0.186 (95% CI: 0.799-0.826). SHAP plots demonstrated that tumor size was the most vital risk factor for LNM. In both training and test sets, Xgboost had better AUC (0.761 vs 0.745; 0.813 vs 0.775; respectively), and it also achieved a smaller Brier score (0.202 vs 0.204; 0.186 vs 0.191; 0.220 vs 0.221; respectively) than the nomogram model based on LR in those three different sets. After adjusting for five most influential variables (tumor size, age, ER, HER-2, and PR), prediction score based on the Xgboost model was still correlated with LNM (adjusted OR:2.73, 95% CI: 1.30-5.71, P=0.008).

**Conclusions:**

The Xgboost model outperforms the traditional LR-based nomogram model in predicting the LNM of IMPC patients. Combined with SHAP, it can more intuitively reflect the influence of different variables on the LNM. The tumor size was the most important risk factor of LNM for breast IMPC patients. The prediction score obtained by the Xgboost model could be a good indicator for LNM.

## Introduction

Invasive micropapillary carcinoma (IMPC), a special subtype of invasive breast cancer, was classified as a new histological type by the World Health Organization (WHO) in 2003 (1). Since Fisher et al. (2) first reported invasive papillary carcinoma with morula-like morphologic changes in 1980, there have been different reports on the pathological diagnostic criteria of IMPC. In all invasive breast cancers, the reported incidence of IMPC varies greatly from 2.0% to 8.0% (1), which is mainly because IMPC is most often part of invasive ductal carcinoma morphology, rather than the entirety of cancer.

Unlike invasive ductal carcinoma, patients with IMPC have a higher incidence of lymph node metastasis (LNM) and a shorter survival time (3–5). It has been known that LNM is correlated with a worse prognosis for breast cancer patients (6). Preoperative assessment of axillary lymph node metastasis can help physicians to implement some interventions such as neoadjuvant chemotherapy in advance, so that patients could benefit from individualized regimens. Regrettably, only core needle biopsy can provide the most direct evidence of lymph node metastasis, but it is expensive and time-consuming. Therefore, it is vital to develop an accurate and convenient model to evaluate the status of axillary lymph node metastasis.

Recently, Ye et al. constructed a nomogram to predict preoperative lymph node involvement of breast IMPC (7), but this LR-based model can only give low area under curve (AUC) of 0.735. Besides, the absence of external validation and the comparison of different models limit the application of the nomogram model. For the past few years, machine learning (ML) has drawn wide attention and has been applied to solve various medical problems, including outcome prediction and treatment support (8–10). Although ML has also been used to predict axillary lymph node metastasis in breast cancer (11, 12). it has not been used in IMPC. Besides, even with huge samples, these ML models lacked concrete explanations and intuitional understanding, limiting their wider applications. To solve the problem, SHapley Additive exPlanations (SHAP) framework, which was firstly proposed by Lundberg et al. (13) and is able to evaluate the contribution of each explanatory variable in any ML models (14), was introduced into this study.

This study aims to develop different ML models to predict axillary lymph node metastasis of breast IMPC and compare the predictive ability of different models. Furthermore, the SHAP framework was applied to intuitively explain the performance of the optimal model. Besides, the risk factors of LNM were also been explored.

## Methods and patients

### Patient selection

In this retrospective analysis, a total of 1405 patients diagnosed with breast IMPC ((ICD-O-3 8507) from Surveillance, Epidemiology, and End Results (SEER) database from 2010 to 2015 were incorporated for ML models construction; and 142 patients diagnosed with breast IMPC from Harbin Medical University Cancer Hospital between 2010-2015 were included for the external validation of the optimal ML model. In every state of the United States, cancer is a reportable disease, so no informed patient consent was required to release the SEER database. The ethics committee of Harbin Medical University Cancer Hospital approved this study. It complies with the World Medical Association Declaration of Helsinki in 1964 and subsequently amended versions. An informed consent form was signed prior to undergoing treatment.

Inclusion criteria (1): pathologically confirmed breast IMPC ((ICD-O-3 8507) (2); unilateral breast IMPC (3); patients diagnosed between 2010-2015; and (4) all patients in the external validation cohort underwent surgery in our hospital.

Exclusion criteria (1): bilateral, single primary breast IMPC; and (2) breast subtype record not available or unknown.

The flow chart for patient selection is shown in **Figure S1**.

### Study outcome

The primary endpoint of this study was axillary lymph node metastasis. If the pathologist examines one or more axillary lymph nodes to be positive, then the axillary lymph node metastasis is confirmed.

### Feature selection and data preprocessing

The method of KNNImputer was applied to variables with a missing age percentage of less than 30% (15). Features statistically correlated with LNM in univariable analysis were selected to develop ML models (**Table 1**). Notably, because the external validation cohort lacked male samples, gender features were excluded for model stability. Besides, other features, including estrogen receptor (ER), progesterone receptor (PR), human epidermal growth factor receptor2 (HER-2) and laterality (16–20), which had been proved to be related with LNM, were incorporated for model construction.

**Table 1:** Clinical and pathological characteristics of different cohorts.

Variable	SEER Cohort				External Validation Cohort			
		Non-LNM	LNM	p		Non-LNM	LNM	p
	N=1405 (%)	n=687 (%)	n=718 (%)		N=142 (%)	n=47 (%)	n=95 (%)	
**Age**	62 [52, 71]	64 [55, 73]	59 [49, 69]	**<0.001**	52.69 (10.22)	56.13 (9.69)	50.99 (10.10)	**0.004**
**Sex**				**0.027**				NA
female	1378 (98.1)	680 (99.0)	698 (97.2)		142 (100.0)	47 (100.0)	95 (100.0)	
male	27 (1.9)	7 (1.0)	20 (2.8)		NA	NA	NA	
**Laterality**				0.905				0.332
left	690 (49.1)	339 (49.3)	351 (48.9)		81 (57.0)	30 (63.8)	51 (53.7)	
right	715 (50.9)	348 (50.7)	367 (51.1)		61 (43.0)	17 (36.2)	44 (46.3)	
**Subtype**				0.134				0.888
luminal A	1042 (74.2)	526 (76.6)	516 (71.9)		51 (35.9)	16 (34.0)	35 (36.8)	
luminal B	242 (17.2)	111 (16.2)	131 (18.2)		91 (64.1)	31 (66.0)	60 (63.2)	
HER-2 OE	64 (4.6)	29 (4.2)	35 (4.9)		NA	NA	NA	
TNBC	57 (4.1)	21 (3.1)	36 (5.0)		NA	NA	NA	
**ER**				0.061				1
negative	128 (9.1)	52 (7.6)	76 (10.6)		3 (2.1)	1 (2.1)	2 (2.1)	
positive	1277 (90.9)	635 (92.4)	642 (89.4)		139 (97.9)	46 (97.9)	93 (97.9)	
PR				0.837				0.426
negative	274 (19.5)	136 (19.8)	138 (19.2)		17 (12.0)	4 (8.5)	13 (13.7)	
positive	1131 (80.5)	551 (80.2)	580 (80.8)		125 (88.0)	43 (91.5)	82 (86.3)	
**HER-2**				0.238				0.951
negative	1099 (78.2)	547 (79.6)	552 (76.9)		122 (85.9)	41 (87.2)	81 (85.3)	
positive	306 (21.8)	140 (20.4)	166 (23.1)		20 (14.1)	6 (12.8)	14 (14.7)	
**Tumor Size**				**<0.001**				**0.003**
<=20 mm	793 (56.4)	532 (77.4)	261 (36.4)		73 (51.4)	33 (70.2)	40 (42.1)	
20-50 mm	469 (33.4)	142 (20.7)	327 (45.5)		67 (47.2)	14 (29.8)	53 (55.8)	
>50 mm	143 (10.2)	13 (1.9)	130 (18.1)		2 (1.4)	0 (0.0)	2 (2.1)	

LNM, lymph node metastasis; HER2, human epidermal growth factor receptor2; TNBC, triple negative breast cancer; ER, estrogen receptor; PR, progesterone receptor. The bold values/numbers mean: p value < 0.05. NA, Not Available.

### The development of ML models

**Figure 1:**
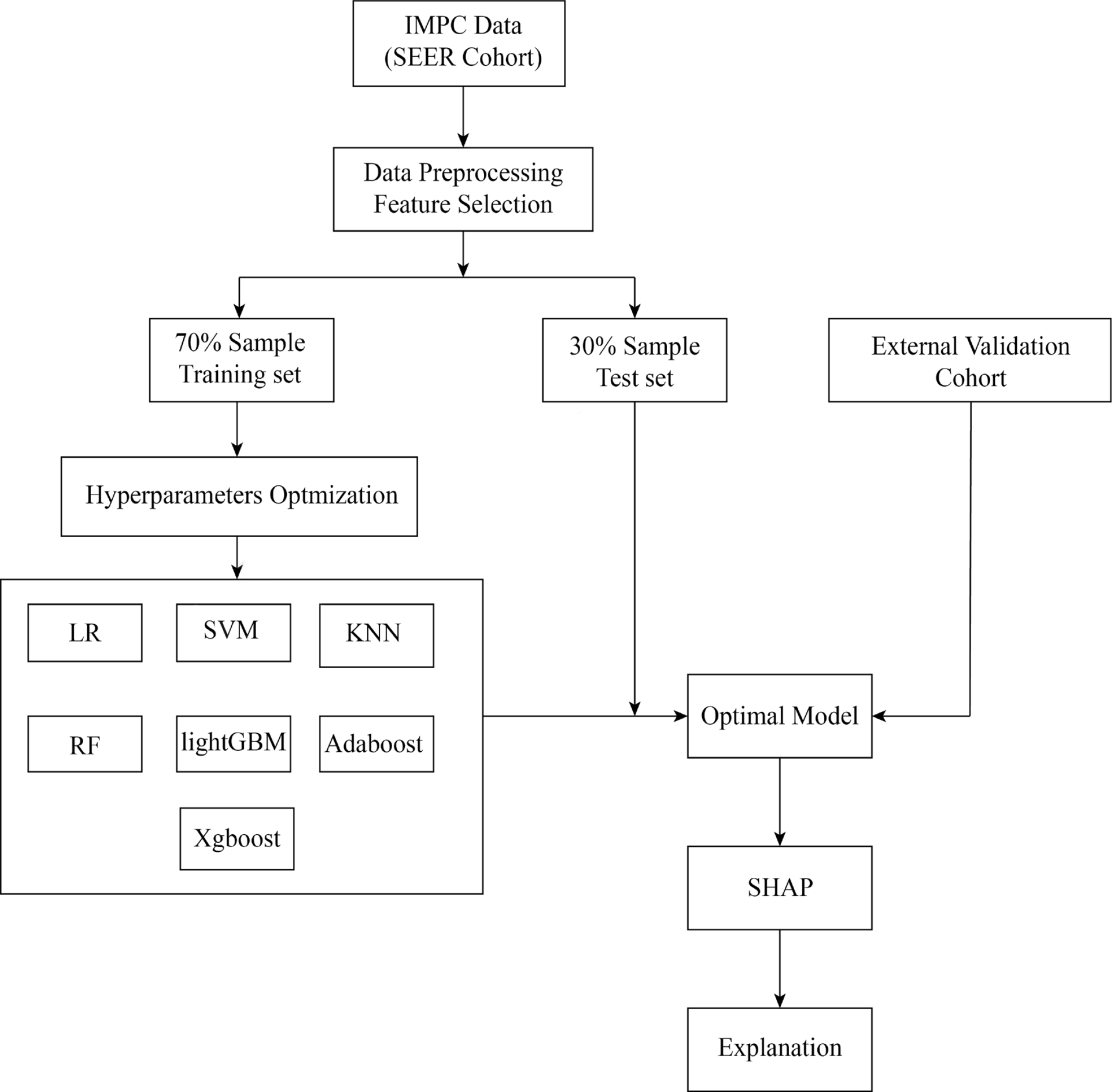
Flow chart for the development, explanation and validation of models.

We introduced seven ML algorithms using clinical and pathological data to predict axillary LNM, and these algorithms are LR, support vector machine (SVM), k-­nearest neighbor (KNN), random forest (RF), Light Gradient Boosting Machine (lightGBM), adaptive boosting (AdaBoost) and extreme gradient boosting (XGBoost). LR models are commonly used to study the impact of trait variables on a binary classification variable (21). Based on hyperspace, SVM is often used to classify things with multidimensional properties into two categories (22). The KNN system, one of the most commonly used nonparametric classification techniques, works on the premise that if the k-nearest samples in the vicinity of a sample mostly belong to a certain class in the feature space, they must also belong to the same category (23). A classifier that uses multiple trees for training and predicting samples is known as the RF, which reduces training variance and improves integration and generalization (24). The Microsoft LightGBM is an ensemble algorithm that implements gradient boosting efficiently (25). AdaBoost, a powerful ensemble method, is an ensemble of weak learners that improves generalization ability (26). XGBoost is a machine learning technology that can efficiently and flexibly process missing data and build accurate prediction models with weak prediction models (27). All the patients were randomly divided into two groups (training set and test set) in a ratio of 7:3. The ML model hyperparameters are optimized with ten-fold CV grid search. The training set was applied to construct ML models. The test set cohort was applied to evaluate the performance of different ML models. In order to avoid over-fitting and improve the prediction ability of the model, the hold-out method was applied. External validation cohort was used to validate the performance of the optimal ML model (**Figure 1**).

### The interpretability of optimal ML model

ML models are often regarded as ‘black boxes’ because it is difficult to explain why they can accurately predict the special cohort of patients. Therefore, we bring in the SHAP value to determine the optimal ML model in this research. SHAP is a new method to explain the contribution of different variable in any ML models (14). Its interpretability performance had been validated in many cancers (28–31). In contrast to other methods, the SHAP method is based on sound theoretical groundwork, providing both local and global interpretability (32). We used SHAP values to assess the probability of LNM of whole cohort or an individual.

### Statistical analysis

All the analysis were conducted by R software version 4.1.3 (forestmodel and dplyr packages) and python version 3.9.7 (scikitplot, sklearn, matplotlib.pyplot, lightgbm, xgboost, sklearn.neighbors, sklearn.svm, numpy, and shap packages).

Frequencies and percentages (%) were applied to describe categorical variables, while the chi-squared test or Fisher’s exact test was applied to assess differences. The median and mean values of continuous variables were presented with the interquartile range (IQR) and standard deviation (SD). The AUC was applied to compare the performance of each ML model. The Brier score (33) was applied to evaluate the calibration of each ML model. The best cut-off value was determined by Youden’s index. Multivariable analysis was conducted by LR. A nomogram was established on the basis of multivariate analysis, and a graphic analysis was performed on the differences between actual and predicted probabilities obtained by the nomograms. P<0.05 was deemed statistically significant.

## Results

### The baseline of breast IMPC patients

The SEER cohort included 1405 breast IMPC patients, 718 (51.1%) of whom suffered from LNM, the external validation cohort covered 142 breast IMPC patients, 95 (66.9%) of whom suffered from LNM, and most patients were female and belonged to luminal subtype in both cohorts. Besides, the patients among the SEER cohort and external validation cohort who belonged to ER accounted for respectively 90.9% and 97.9%, the ones belong to PR accounted for respectively 80.5% and 88.0%, while those diagnosed with HER-2 positive were 306 (21.8%), and 20 (14.1%), respectively.

The association between age and tumor size with LNM was observed in both cohorts (P <0.05). The relation between sex and LNM was confirmed in SEER cohort, while remaining untouched in external validation cohort because of the limited samples. (**Table 1**)

### The predictive ability of different ML models

**Figure 2:**
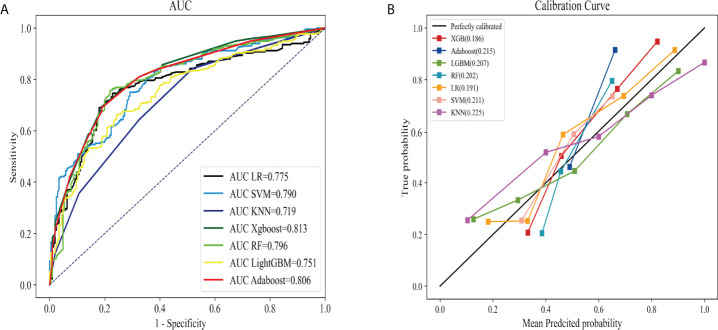
The perfomance comparison of different machine learning models in predicting lyph node metastasis. The receiver operating characteristic curves **(A)** and calibration curves **(B)** of different models.

AUC and Brier score were adopted to compare seven ML models, revealing that model Xgboost outperformed with the biggest AUC of 0.813 (95% CI: 0.7994 - 0.8262; **Figure 2A**), the calibration curve (the red line) that was closest to the perfectly calibrated curve (the black line), and the smallest Brier score of 0.186 (95% CI: 0.799-0.826; **Figure 2B**). Therefore, model Xgboost was selected to predict LNM of IMPC.

### The visualization of feature importance

**Figure 3:**
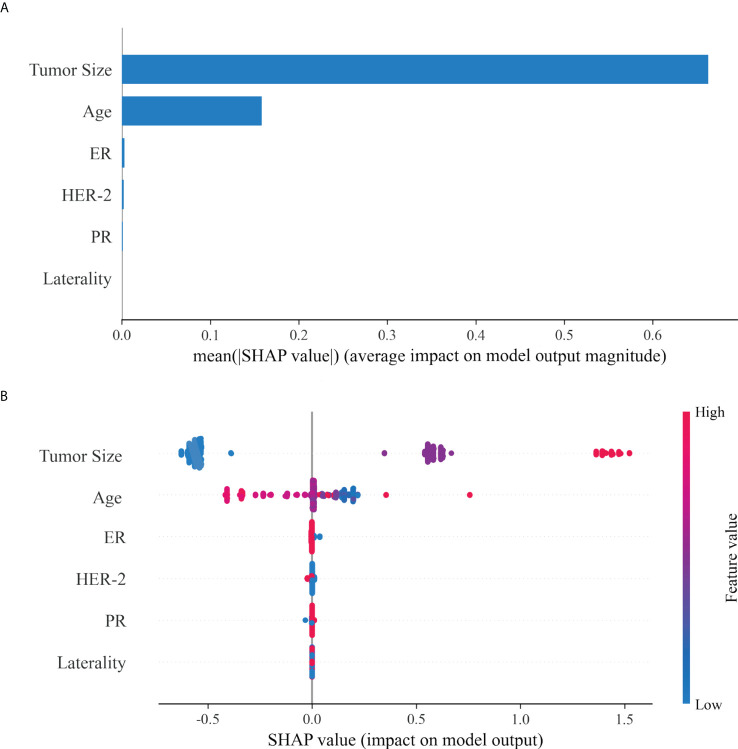
The interpretation of optimal model (Xgboost). **(A)**: The importance ranking of different variables according to the mean (|SHAP value|); **(B)**: The importance ranking of different risk factors with stability and interpretation using the optimal model. The higher SHAP value of a feature is given, the higher risk of lymph node metastasis the patient would have.The red part in feature value represents higher feature value.

SHAP was adopted to evaluate the effect of these selected variables on the LNM of IMPC, and to explain such variables. The feature importance of variables was ranked through the mean (|SHAP value|), and the tumor size stood out (**Figure 3A**). **Figure 3B** illustrated their detailed impact on LNM. The SHAP value (x-axis) referred to how the value or status of different variables influenced the LNM in the model, while the feature value (y-axis) the change of a certain variable. A bigger tumor size and smaller age increased the risk of LNM, while the status of ER, HER-2, PR and laterality exerted limited impact.

### Molecular subtype-based analysis

**Figure 4:**
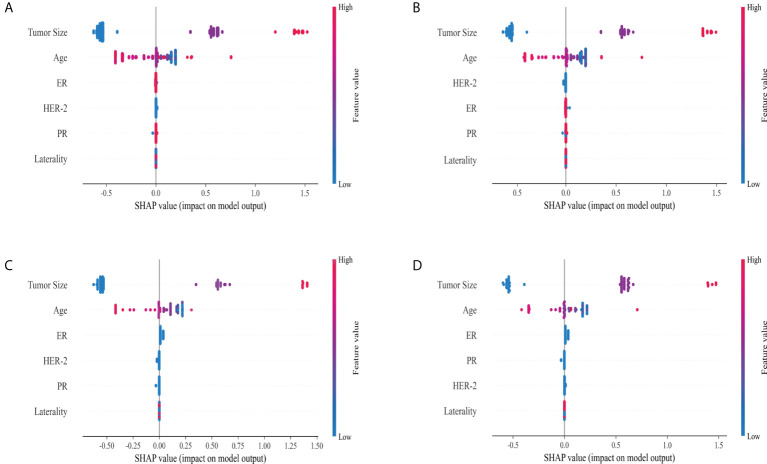
Variable importance in ML classification for Luminal A (**A**, n = 1042), Luminal B (**B**, n = 242), HER-2 overexpression (**C**, n = 64) and TNBC (**D**, n=57).

Tumor size and age served as important risk factors for LNM in different molecular subtype of breast IMPC. ER status was the third important risk factor for LNM in luminal A, HER-2 OE, and TNBC subtypes, while HER-2 was the third in luminal B subtype. (**Figure 4**)

### Individualized prediction

**Figure 5:**
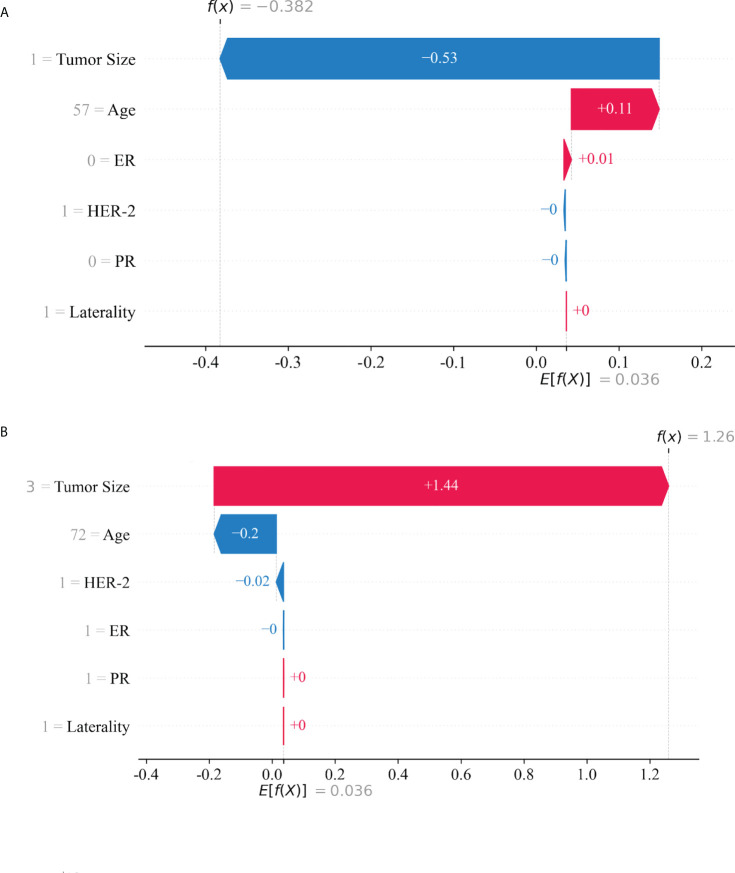
The interpretation of model prediction results with the two samples. A patient with no lymph node metastasis **(A)**. A patient with lymph node metastasis **(B)**.

Based on the SHAP value, the risk of LNM in each patient was calculated. Two classical patients, including a 57-year-old without LNM and a 72-year-old with LNM, were explored to interpret the optimal model (**Figure 5**). The waterfall plot demonstrated the impact of variables on LNM, in which the red arrow indicated the increased risk, while the blue arrow the decreased risk. The SHAP value was calculated by combining the effects of variables, which corresponded to the prediction score. The non-LNM patient (**Figure 5A**) performed a low SHAP value (-0.382) and prediction score (0.405529), and the LNM patient (**Figure 5B**) exhibited a high SHAP value (1.26) and prediction score (0.778945).

### The multivariable logistic regression analysis

**Figure 6:**
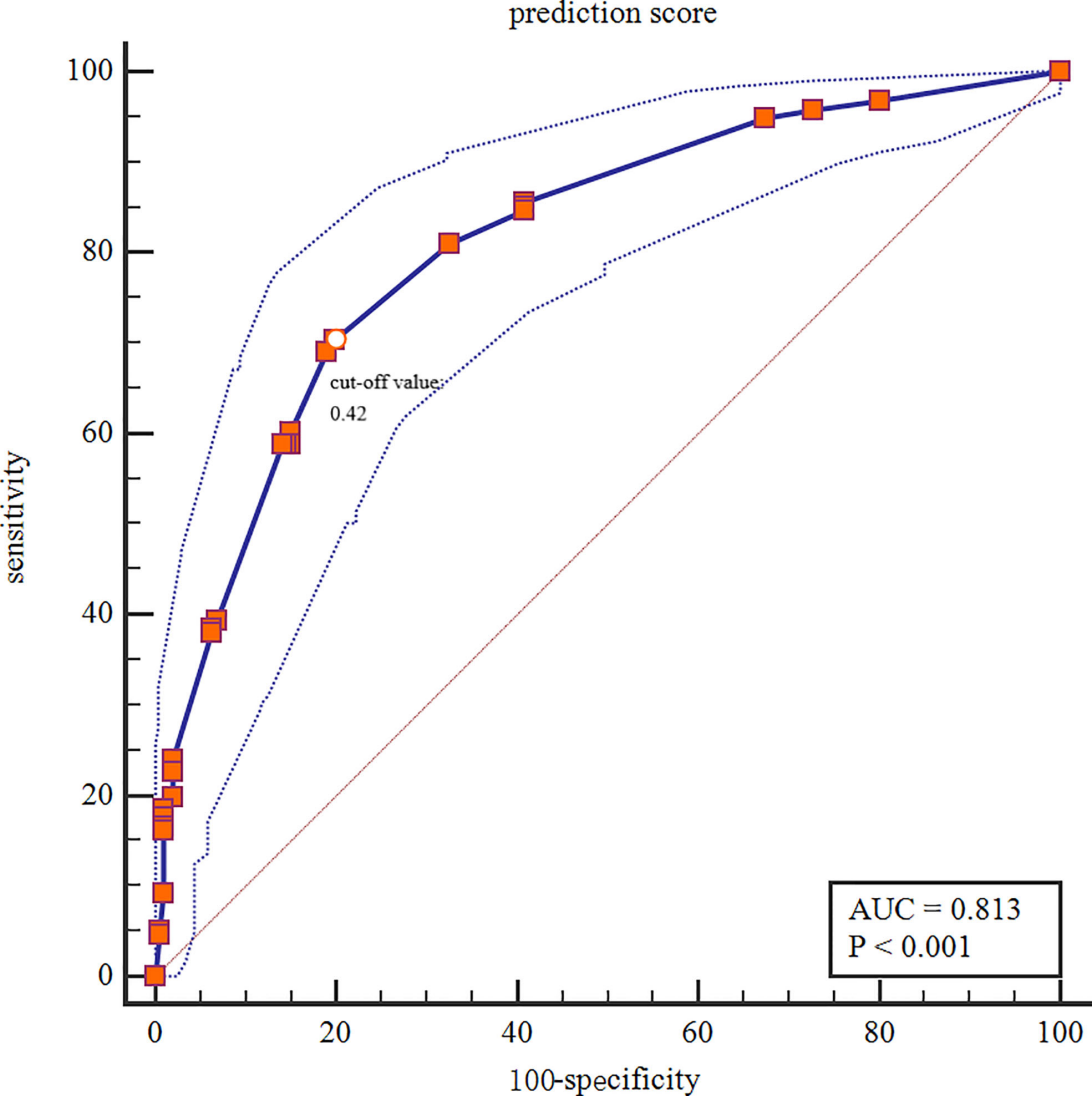
Categorization threshold of Prediction score.

**Figure 7:**
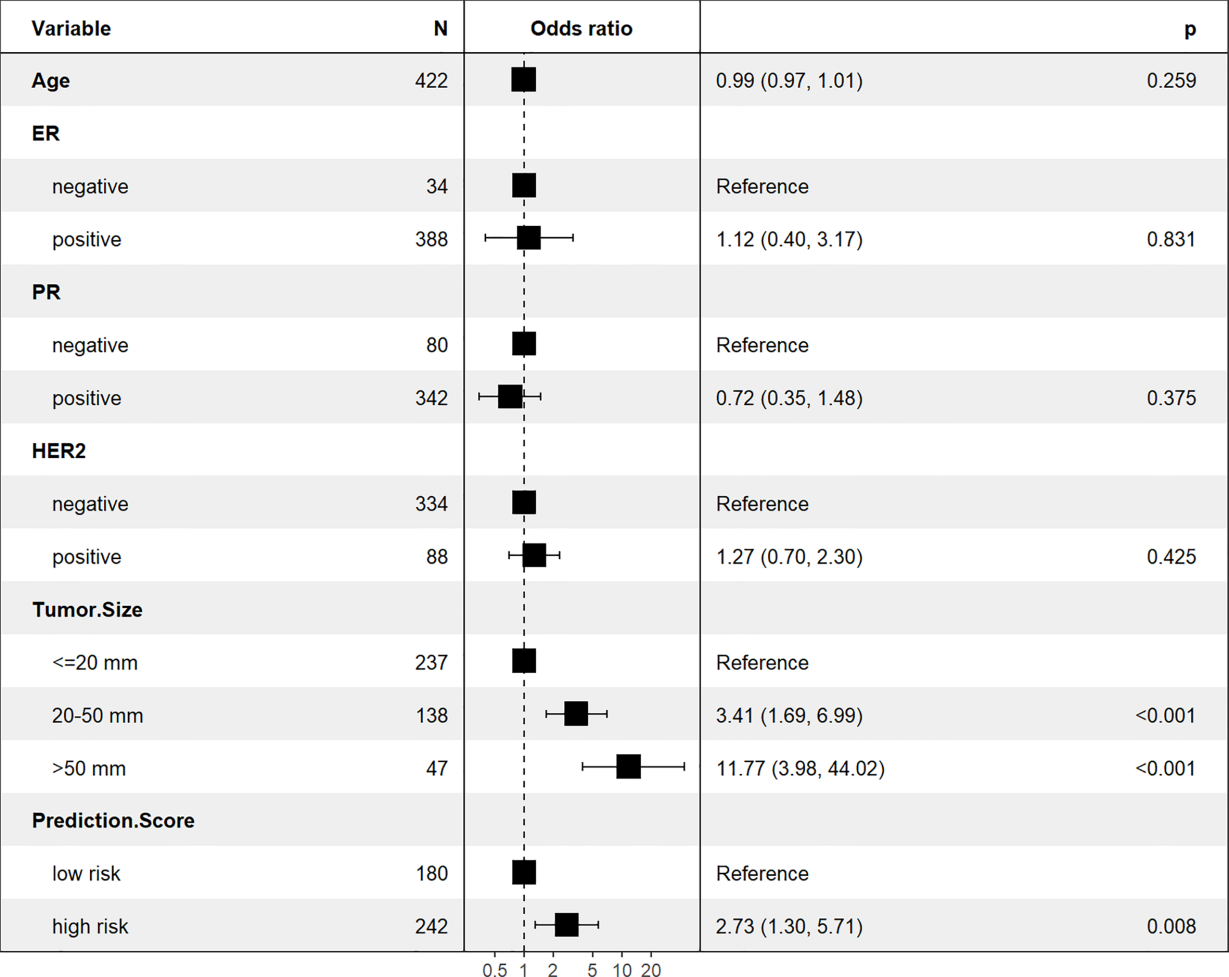
The multivariable logistic regression analysis for LNM prediction.

The Xgboost model was applied to predict LNM in the test set. All patients were divided into high and low risk groups according to the best cut-off value (0.42) determined by the Youden’s index (**Figure 6)**. The unadjusted LR analysis found that patients in the high-risk group were more prone to LNM (unadjusted OR:8.86, 95% CI: 5.71-13.99, P<0.001). Despite the adjustment of the five most influential variables (tumor size, age, ER, HER-2, and PR), prediction score was correlated with LNM (adjusted OR:2.73, 95% CI: 1.30-5.71, P=0.008; **Figure 7**).

### The external validation for the predictive model

**Figure 8:**
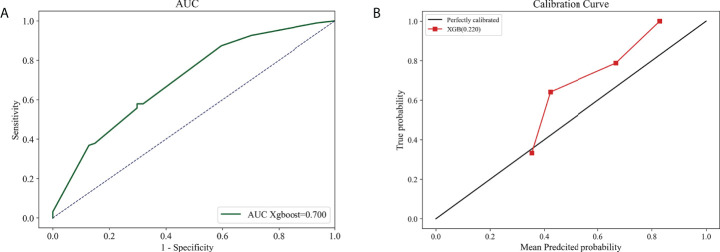
The external validation based on Xgboost model. The AUC curve **(A)** and calibration curve **(B)** 1n external validation cohort.

The Xgboost model, which outperformed in stability and accuracy compared with other ML models, was assessed by employing 142 breast IMPC samples from our hospital, so as to further identify its accuracy and stability. The result demonstrated that the model achieved a big AUC of 0.700 (95% CI: 0.682 - 0.72; **Figure 8A**), and a low Brier score of 0.220 (95% CI: 0.216-0.225; **Figure 8B**).

### The performance of comparison of Xgboost and nomogram (LR) model

A nomogram was constructed in train set, test set, and external validation cohort, respectively, according to LR modes (**Figure S2**). All three nomograms based on clinical and pathological variables performed favorably. Nevertheless, the model Xgboost exhibited a bigger AUC in training (0.761 vs 0.745) and test sets (0.813 vs 0.775) compared with the LR model. The AUCs of these two models were similar (0.700 vs 0.703) in external validation cohort. Besides, Brier Score of Xgboost was smaller in these three sets (0.202 vs 0.204; 0.186 vs 0.191; 0.220 vs 0.221; respectively; **Table 2**).

**Table 2:** The comparison of Xgboost and nomogram (LR) model.

Model	Training set		Test set		External validation cohort	
	AUC	Brier Score	AUC	Brier Score	AUC	Brier Score
LR	0.745 (0.730-0.758)	0.204 (0.199-0.210)	0.775 (0.761-0.790)	0.191 (0.186-0.197)	0.703 (0.685-0.718)	0.221 (0.215-0.225)
Xgboost	0.761 (0.746-0.776)	0.202 (0.197-0.206)	0.813 (0.799-0.826)	0.186 (0.182-0.190)	0.700 (0.683- 0.716)	0.220 (0.216-0.225)

## Discussion

As a special subtype of breast cancer, IMPC cells was susceptible to invasion and metastasis because of special growth pattern and histological morphology induced by polarity reversal (34). Compared to breast invasive ductal carcinoma (IDC), breast IMPC had higher LNM rate and worse survival outcome (4, 35–37). Given the close association between LNM and survival outcome, a tool that identifies LNM can help doctors in instituting heal project and timely adjusting the treatment program. This paper chose the best ML model Xgboost following the comparison of seven powerful ML models to predict LNM of breast IMPC, whose performance was validated in the test set and external validation cohort. Through the SHAP values and plots, the feature importance rank and contribution to LNM of risk factors were intuitively demonstrated. Besides, the prediction score based on Xgboost was proved to be an independent predictive factor for LNM.

Nassar et al. found no significant differences in lymph node status, ER status, tumor size, grade, or lymph vascular invasion between tumors with different invasive micropapillary components (5). In addition, the difference of survival outcome between IMPC and IDC with similar stage was negligible. Therefore, despite their worse survival outcome than IDC patients, IMPC patients follow IDC treatment protocols, the current standard of care (38).

The correlation between LNM and worse survival time of breast cancer patients is known (6). Breast cancer patients with LNM underwent axillary lymph node dissection (ALND) in the past. The results of ACOSOG Z0011 (Alliance) Randomized Clinical Trial, however, indicated the similar 10-year overall survival between patients treated with ALND and those treated with sentinel lymph node dissection (SLNB) alone in T1 or T2 stage with 1 or 2 SLN metastasis (39), which explained the current wide application of SLNB for early operable invasive breast cancer patients with negative clinical lymph node. Nevertheless, it was still controversial if SLNB was suitable for breast IMPC (40). The information about the status of axillary lymph node facilitated doctors in developing an individualized treatment plan, thus avoiding overtreatment or undertreatment, which highlighted that the management of axillary lymph node deserved more attention.

In response, Ye and his team developed a nomogram to predict preoperative lymph node involvement for breast IMPC patients (7), and propose nomogram as a good tool for LNM prediction. Their study based on SEER database, however, lacked external validation and the comparison of model performance. Actually, the performance comparison between nomogram and ML models had been conducted in different disease. Rasheed et al. proved the higher accuracy of boosted decision tree than nomogram in predicting overall survival among patients with tongue cancer (41), and Thara and his team demonstrated the bigger AUC of random forest classifier model than nomogram in predicting intracranial injury following cranial CT of the brain (42), which unfortunately were also short of external validation and intuitive explanation to the model.

Previous studies took that most breast IMPC were ER positive (72%-75%), almost half were HR positive, and patients with HER-2 positive ranged from 10%-30% (43–45). In this paper, the proportion of patients in the SEER cohort and external validation cohort with ER positive was 90.9% and 97.9%, respectively, that with PR positive was respectively 80.5% and 88.0%, while that with HER-2 positive was respectively 21.8% and 14.1%, which shared the results of the above studies, and verified the stability and reliability of the samples adopted. Training set was adopted to develop the ML models, and the ability of optimal model Xgboost and nomogram in test set and external validation cohort was compared, demonstrating the bigger AUC of model Xgboost in training (0.761 vs 0.745) and test sets (0.813 vs 0.775), and the smallest Brier Score of Xgboost in three sets (0.202 vs 0.204; 0.186 vs 0.191; 0.220 vs 0.221; respectively; **Table 2**). The AUC of Xgboost was slightly less than that of LR model (nomogram) in external validation cohort, which came down to small sample and racial difference (all patients in external validation cohort were Chinses while most patients in training and test sets came from US), but the Xgboost was still a better model than nomogram based on LR. Meanwhile, instead of nomogram which only showed the score of each variable in predicting LNM, SHAP was adopted in the paper to visually demonstrate the contribution of each variable. The SHAP plots intuitively displayed the increased or decreased contribution of each variable to LNM, and the bigger SHAP value indicated higher probability of LNM. In addition, SHAP values indicated the feature importance rank of each variable, and tumor size was the most influential risk factor for LNM. The feature importance of each variable in different molecular subtype was also compared, revealing tumor size to be the most important one. Instead, the application of nomogram failed to rank the importance of features, which validated the better practicability and predictive ability of model Xgboost. The contribution of prediction score was also evaluated based on Xgboost. After adjusting for confounding factors, prediction score was significantly associated with LNM, and patients in high prediction score group had higher risk for LNM. ML model was generally a better tool than nomogram based on LR in predicting LNM of breast IMPC patients.

Despite being the first to predict LNM of breast IMPC patients using ML models and compare its performance with nomogram based on LR to the authors’ knowledge, this study was limited in the following aspects. Firstly, a prospective analysis was required to further identify the performance of Xgboost model even for the paper, a multicenter retrospective analysis. Secondly, the huge samples from SEER database could not make up for its limited clinical and pathological information, which required a cohort including more details of breast IMPC patients. Besides, the XGBoost model combined with more features (like Grade) could train more useful information about LNM, so as to promote its performance, which consolidated its clinical advantages compared with LR model. Thirdly, the clinical application of the ML model constructed based on SEER database was limited due to the highly homogenous feature of IMPC, a rare subtype of invasive breast cancer. Therefore, a larger sample contained different histological types of breast cancer, like breast invasive ductal cancer, was needed to expand the clinical practicability of the best ML model.

## Conclusions

The ML models, especially Xgboost, outperformed traditional LR-based nomogram model in predicting LNM of breast IMPC patients. The combination of Xgboost and SHAP intuitively reflected the influence of different variables on LNM, and the tumor size was the most important risk factor of LNM for breast IMPC patients. In addition, the prediction score derived from Xgboost model served as a good indicator for LNM.

## Data availability statement

The raw data supporting the conclusions of this article will be made available by the authors, without undue reservation.

## Ethics statement

This research was approved by the ethics committee of Harbin Medical University Cancer Hospital. It complies with the World Medical Association Declaration of Helsinki in 1964 and its later amendments. All patients signed the informed consent before each treatment.

## Author contributions

CJ and YH conceptualized and designed the work. YX, KQ and XY collected all the data. CJ and SZ drafted and analyzed the manuscript. All authors contributed to the article and approved the submitted version.

## Funding

This work was supported by the Haiyan Foundation of Harbin Medical University Cancer Hospital (Grant Number: JJQN2022-01). The funder played no role in the study design, data collection and analysis, decision to publish, or preparation of the manuscript.

## Acknowledgments

Thanks for the data provided by Harbin Medical University Cancer Hospital.

## Conflict of interest

The authors declare that the research was conducted in the absence of any commercial or financial relationships that could be construed as a potential conflict of interest.

## Publisher’s note

All claims expressed in this article are solely those of the authors and do not necessarily represent those of their affiliated organizations, or those of the publisher, the editors and the reviewers. Any product that may be evaluated in this article, or claim that may be made by its manufacturer, is not guaranteed or endorsed by the publisher.
