# Supplementary figures and images for: Prediction of lymph node metastasis in patients with breast invasive micropapillary carcinoma based on machine learning and SHapley Additive exPlanations framework
Source: Front Oncol. 2022 Sep 15;12:981059. doi: 10.3389/fonc.2022.981059 (PMC9520536; doi:10.3389/fonc.2022.981059)

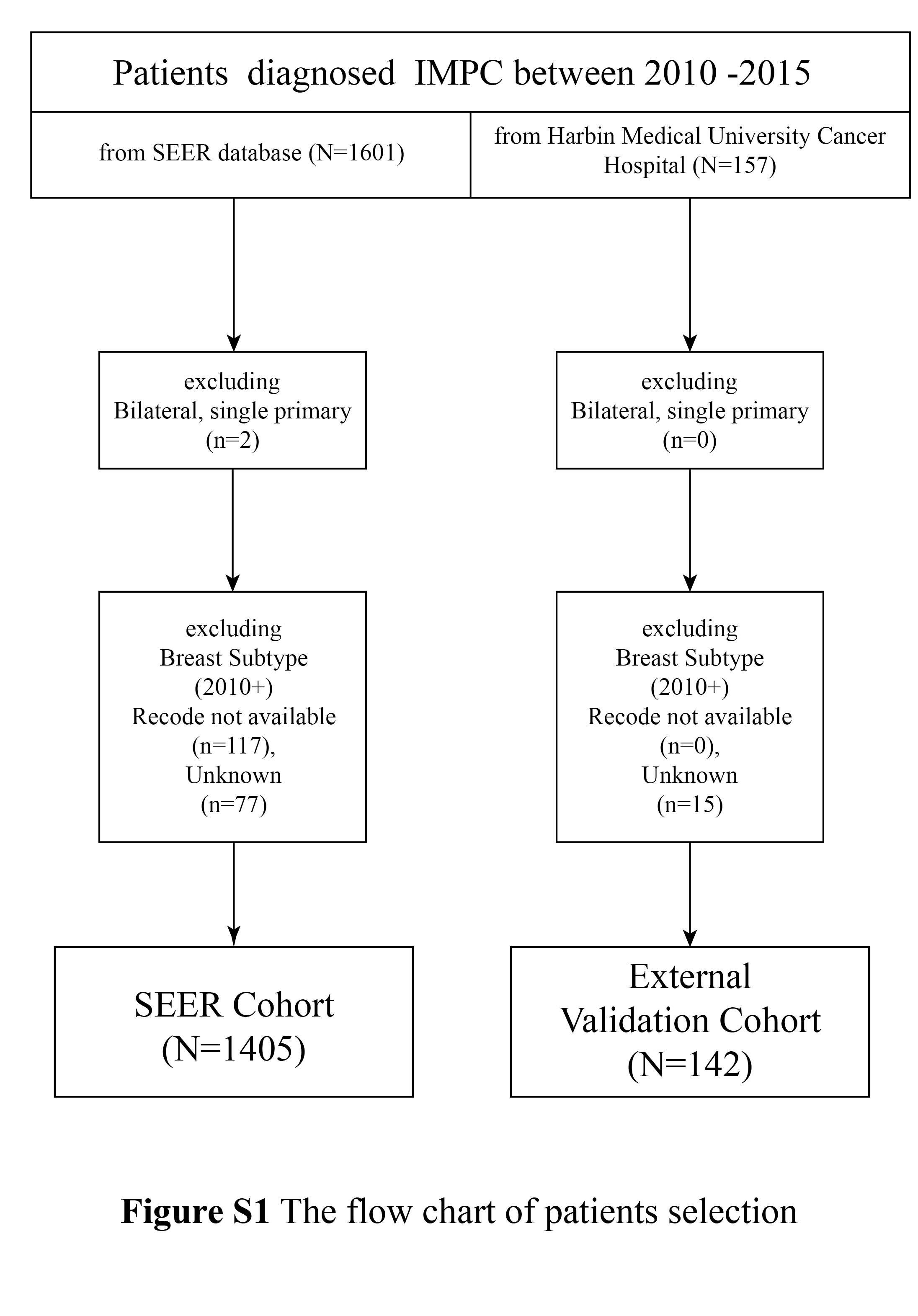

Supplement: Supplementary file 1 [file Image_1.png]
